# 968 Cool Running Water in the Prehospital Environment: Implementation Challenges and Future Opportunities

**DOI:** 10.1093/jbcr/iraf019.499

**Published:** 2025-04-01

**Authors:** Kevin Mackey, Maleea Holbert, Bronwyn Griffin

**Affiliations:** Sacramento Fire Department; Queensland Children’s Hospital and Griffith University; Griffith University

## Abstract

**Introduction:**

Providing immediate and appropriate first aid for thermal burn injuries improves clinical outcomes for patients, reducing burn depth progression and the likelihood of requiring a skin graft. The application of 20 minutes of cool running water (20CRW) within the first three hours post-burn has become standard first aid for thermal injuries across the United Kingdom, Europe, and Australia. We present a qualitative investigation of prehospital first responders during the implementation of 20CRW into burn first aid guidelines and protocols.

**Methods:**

A semi-structured focus group interview was conducted with a team of first responders from one station in April 2024 – two months after the implementation of 20CRW launched into prehospital guidelines. We aimed to explore first responders’ early experiences of providing this first aid treatment in the prehospital environment; gain insights into the cultural transition from load-and-go to remaining on-scene to administer 20CRW; and discuss the use of water when water sources are unpredictable. The focus group interview was recorded and transcribed verbatim. Themes were generated from the transcript using inductive qualitative content analysis.

**Results:**

Twelve prehospital first responders participated in the focus group interview. Data were coded and categorized into 11 themes. Environmental and logistical difficulties included: limited access to clean or cool running water, lack of controlled substances on fire engines, having a single booster line on engines (unable to irrigate multiple anatomical regions or patients at once), and maintaining patient privacy when administering 20CRW in non-residential settings. The most common operational challenge was the concern over prolonged scene time to administer 20CRW, as opposed to traditional immediate transport. This concern was reported by both first responders and the general public. Tank water from fire engines was identified as a viable source of portable water, facilitating on-scene provision of 20CRW in complex settings. First responders also reported that 20CRW provided pain relief for some burn patients. Effective communication and clear explanation of care was identified as facilitators to on-scene first aid treatment, assisting in patient and public compliance. In addition, emergency dispatch instructing patients to commence 20CRW before first responders arrive was reported as a major facilitator.

**Conclusions:**

First responders successfully overcame many perceived barriers to implementing 20CRW in prehospital environments. While operational and logistical challenges remain, they are not insurmountable and provide opportunities for collaboration and creative approaches.

**Applicability of Research to Practice:**

The solutions employed by first responders provide valuable insights that can be scaled to other prehospital care providers, ultimately improving the effectiveness and consistency of 20CRW provision across different settings.

**Funding for the Study:**

This research received competitive government grant funding, awarded to the Principal Investigator.

